# Mutation screening of melatonin-related genes in patients with autism spectrum disorders

**DOI:** 10.1186/1755-8794-3-10

**Published:** 2010-04-08

**Authors:** Lina Jonsson, Elin Ljunggren, Anna Bremer, Christin Pedersen, Mikael Landén, Kent Thuresson, MaiBritt Giacobini, Jonas Melke

**Affiliations:** 1 Institute of Neuroscience and Physiology, Department of Pharmacology, Gothenburg University, Sweden; 2 Department of Molecular Medicine and Surgery, Karolinska Institutet, Stockholm, Sweden; 3 Stockholm Center for Psychiatric Research, Karolinska Institutet, Stockholm, Sweden; 4 Institute of Neuroscience and Physiology, Department of Neurochemistry and Psychiatry, Neuropsychiatric unit of Mölndals hospital at the University of Gothenburg, Sweden

## Abstract

**Background:**

One consistent finding in autism spectrum disorders (ASD) is a decreased level of the pineal gland hormone melatonin and it has recently been demonstrated that this decrease to a large extent is due to low activity of the acetylserotonin O-methyltransferase (ASMT), the last enzyme in the melatonin synthesis pathway. Moreover, mutations in the *ASMT *gene have been identified, including a splice site mutation, that were associated with low ASMT activity and melatonin secretion, suggesting that the low ASMT activity observed in autism is, at least partly, due to variation within the *ASMT *gene.

**Methods:**

In the present study, we have investigated all the genes involved in the melatonin pathway by mutation screening of *AA-NAT *(arylalkylamine N-acetyltransferase), *ASMT, MTNR1A, MTNR1B *(melatonin receptor 1A and 1B) and *GPR50 *(G protein-coupled receptor 50), encoding both synthesis enzymes and the three main receptors of melatonin, in 109 patients with autism spectrum disorders (ASD). A cohort of 188 subjects from the general population was used as a comparison group and was genotyped for the variants identified in the patient sample.

**Results:**

Several rare variants were identified in patients with ASD, including the previously reported splice site mutation in *ASMT *(IVS5+2T>C). Of the variants affecting protein sequence, only the V124I in the *MTNR1B *gene was absent in our comparison group. However, mutations were found in upstream regulatory regions in three of the genes investigated, *ASMT, MTNR1A*, and *MTNR1B*.

**Conclusions:**

Our report of another ASD patient carrying the splice site mutation IVS5+2T>C, in *ASMT *further supports an involvement of this gene in autism. Moreover, our results also suggest that other melatonin related genes might be interesting candidates for further investigation in the search for genes involved in autism spectrum disorders and related neurobehavioral phenotypes. However, further studies of the novel variants identified in this study are warranted to shed light on their potential role in the pathophysiology of these disorders.

## Background

Autism spectrum disorders (ASDs) are pervasive developmental disorders that include Autistic disorder, Asperger syndrome, and pervasive developmental disorder-not otherwise specified (PDD-NOS). These conditions are complex, behaviorally-defined syndromes with variable severity and highly diverse symptomatology and etiologies, characterized by impairments in social interaction and communication and patients often display repetitive behaviors, abnormal movements, and sensory dysfunction [1]. Moreover, the severity of mental retardation (MR) is highly variable among cases with ASD [2].

Twin studies have demonstrated a much higher concordance for ASD in monozygotic (>70%) than in dizygotic twins (0-10%), suggesting genetic factors to play a major role in the etiology [3,4]. However, although the high heritability of autism is well established and inherited forms of the disorder have been demonstrated, most autism cases seem to occur as sporadic cases [5]. Moreover, several monogenic disorders, such as fragile X syndrome, Rett syndrome and tuberous sclerosis, are well known causes of autism like behavior patterns, and recently, rare mutations and copy number variations have been found to be causative or contributory factors for autism spectrum disorders [6-9]. These observations, together with the early age of onset, indicate that rare, high impact, genetic variants may play an important role in the etiology of ASD [5].

Sleep disturbances are often reported in patients with ASD [10], suggesting that neuroendocrine functions involved in the circadian sleep-wake cycle may be altered in these disorders. Moreover, it is well established that mutations altering the biological clock can cause severe sleep problems by modifying sleep phase or duration [11]. Melatonin, a hormone secreted by the pineal gland in the brain, serves as the body's signal for darkness and is involved in various physiologic functions, including sleep induction, circadian rhythm regulation, and immune response [12]. Melatonin as a medication has been shown to improve the sleep of patients with ASD [13,14], suggesting that the endogenous level of this hormone may not be sufficient to adequately set the clock in these patients. Indeed, one consistent biological finding in autism is low levels of melatonin [15-18], and in a recent study, it could be demonstrated that this decrease of melatonin seems to be due to low activity of the acetylserotonin O-methyltransferase (ASMT), the last enzyme in the melatonin synthesis [15].

In the same study, several mutations in the *ASMT *gene were identified, including a splice site mutation, IVS5+2T>C, that were associated with low ASMT activity and melatonin secretion, suggesting that the low ASMT activity in autism is, at least partly, due to variation within the *ASMT *gene. In a replication study investigating almost 400 ASD patients from Italy, Finland, and the UK, several mutations in the *ASMT *gene were identified, including the previously reported splice site mutation and a novel stop-mutation (R319X) [19].

Recently, further support for an involvement of the *ASMT *gene in ASD was presented in a Multiplex Ligation-dependent Probe Amplification (MLPA) study showing that a duplication in the *ASMT *gene was significantly more common in ASD, as compared to controls [20], suggesting that the expression of the ASMT protein may be altered in ASD patients. In addition, other genes implicated in circadian rhythm regulation and central effects of melatonin, *i.e*., the clock genes, have also been found to be associated with autism [21,22]. Taken together, previous data suggest that the *ASMT *gene and the melatonin signaling pathway may play an important role in the etiology of autism spectrum disorders.

The aim of the present study was to search for mutations in genes involved in the melatonin pathway in cases of ASD. To this end, we performed mutation screening of all the genes of the melatonin pathway, i.e., *AA-NAT, ASMT, MTNR1A, MTNR1B *and *GPR50*, in 109 subjects with ASD. Hence, the focus of this study was on identifying rare variants within these genes, rather than on common polymorphisms.

## Methods

### Patient recruitment and clinical assessment

In total, 109 patients with ASD from two different populations were included in this study. In the first population, 65 patients (39 males and 26 females) were recruited at a tertiary neuropsychiatric outpatient unit at Mölndals Hospital, Göteborg, Sweden. Thirty-two of these patients met the criteria for autistic disorder and 33 patients were considered to have pervasive developmental disorder not otherwise specified (PDD-NOS); 24 and 28 of the patients were also diagnosed with moderate and severe mental retardation, respectively. The second patient cohort consisted of 44 ASD patients (30 males and 14 females), recruited from several child and psychiatric outpatient clinics in Stockholm county as well as from the neuropediatric department in Astrid Lindgrens hospital and the neuropediatric department at Sachsska childrens hospital, Stockholm, Sweden. In the second cohort, 26 of the patients met the criteria for autistic disorder, 13 of the patients were considered to have pervasive developmental disorder not otherwise specified (PDD-NOS), 5 met the criteria for Asperger's syndrome, and 21 of the 44 patients had a mental retardation ranging from mild to moderate. All patients from both populations were diagnosed based on clinical evaluation by experienced clinicians using DSM-IV criteria, and the Autism Diagnostic Interview-Revised (ADI-R), and patients diagnosed with medical conditions related to autism and mental retardation, including major chromosomal abnormalities, fragile X syndrome, and tuberous sclerosis, were excluded from the study. The vast majority of included patients were of Swedish origin and the control sample used in this study consisted of 188 (94 male and 94 female) Swedish subjects recruited from the general population in Gothenburg, Sweden [23]. The control group in this study served only to investigate if mutations identified in patients were present also in the general population, hence, the control group was not matched to the patient group with respect to age and gender.

The study was conducted in accordance with the declaration of Helsinki and approved by the local Research Ethics Boards. Written informed consent was obtained from parents or legal guardians, and, if possible (i.e. if the patient did not have severe MR) from patients, as well as from controls.

### DNA analysis

Mutation screening was performed by direct sequencing of the exons, the exon-intron boundaries, the 5' UTR and the proximal promoter of *AA-NAT, ASMT, MTNR1A, MTNR1B*, and *GPR50*. Genotyping of healthy controls for rare variants identified by mutation screening was performed using direct sequencing, pyrosequencing technology (Pyrosequencing AB, Uppsala, Sweden) or RFLP, restriction fragment length polymorphism, (the *MTNR1B *c.-39G, C>A, A) using Cfr3I (Sau96I). Primer sequences and PCR-conditions are summarized in additional file 1 and 2: supplemental table S1 and S2. The ClustalW alignment program http://www.ebi.ac.uk/clustalw/ was used to study conservation of the mutation sites among different species. The possible functional impact of amino acid changes was predicted by the PolyPhen [24](Polymorphism Phenotyping) program http://coot.embl.de/PolyPhen/) which makes predictions based on sequence annotation and alignment as well as structural information and SIFT [25](Sorting Intolerant From Tolerant) http://blocks.fhcrc.org/sift/SIFT.html), which use sequence homology for prediction. Identification of putative binding sites for transcription factors was performed using TFsitescan http://www.ifti.org/cgi-bin/ifti/Tfsitescan.pl and the TRANSFAC database [26].

### Analytical plan

The goal of this study was to search for mutations in genes involved in the melatonin pathway in cases of ASD. To this end, we performed mutation screening of all the genes of the melatonin pathway (*AA-NAT, ASMT, MTNR1A, MTNR1B *and *GPR50*) by direct sequencing. Identified variants were analyzed using different bioinformatic tools (above), however, we did not have the possibility to perform functional analyses in our laboratory. Moreover, to assess if identified variants were present also in the general population, we genotyped a reference group of subjects recruited from the general population in the same geographical area as the patients. However, since our main goal was to study rare variants, rather than common polymorphisms, and since our samples are too small for meaningful statistical comparisons, we did not perform any case-control association studies using the genotype data of common polymorphisms identified in the mutation screening.

## Results

**Figure 1:**
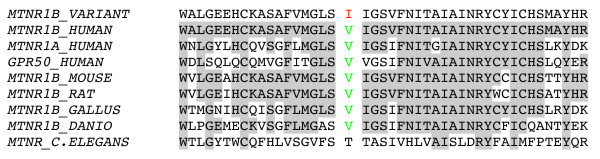
**Peptide sequence alignment of a portion of the MTNR1B-protein from different species (orthologous to the human residues 106-150), and from the human paralogues MTNR1A and GPR50**. Conservation of the Valine 124 residue is indicated in green.

**Figure 2:**
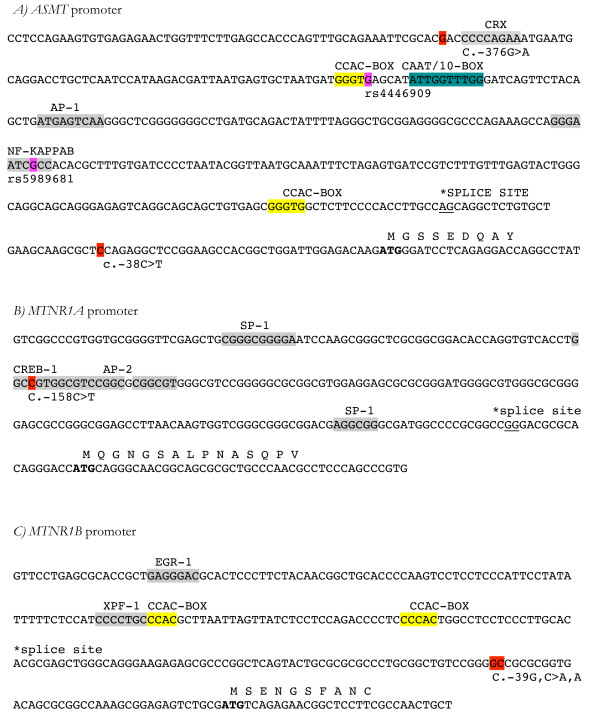
**Sequence and variations located in the promoter sequences of the *ASMT *(A), *MTNR1A *(B), *andthe MTNR1B *(C) genes**. Rare variants identified in ASD patients in our screen are indicated in red. Putative binding sites for major known transcription factors and transcription factor binding sites close to identified mutations are indicated. CCAC-boxes are indicated in yellow and the CAAT-BOX in *ASMT *is indicated in dark green. As reported previously, TATA-boxes are not present in any of the promoter regions examined. The two SNPs (rs4446909 and rs5989681) in *ASMT *previously associated [15] with autism and low transcript levels are indicated in pink.

Six rare variants in the investigated genes were found during the screen (Table 1). The previously reported splice site mutation, IVS5+2T>C, in *ASMT *[15,19], was in our study identified in one patient with autistic disorder and severe mental retardation. Of the identified mutations, only the V124I variant in *MTNR1B *(Table 1) gives rise to an amino acid change. Our analyses using ClustalW revealed that this valine residue at amino acid position 124 is conserved through all vertebrates, and also between MTNR1A and MTNR1B (Figure 1). However, computer based prediction of the functional effect this mutation, using PolyPhen [24] and SIFT [25], revealed negative results (Table 1). Moreover, mutations were found upstream of the transcription start in three of the genes investigated. In *ASMT*, the c.-376G>A variant (also found in one control) is located in close vicinity to a binding site for CRX (Figure 2A) and the c.-158C>T variant (not present in controls) in *MTNR1A *potentially alter the binding of the transcription factors CREB1 and AP-2 (Figure 2B). In addition, two variants that were not present in the control group, c.-38C>T in *ASMT *and c.-39G, C>A, A in *MTNR1B*, are located in the 5'UTR region, hence, potentially altering the translation efficiency of the mRNA and thereby the expression of the protein encoding it (Figure 2A and 2C, respectively).

**Table 1:** Sequence variants identified in *ASMT, MTNR1A, MTNR1B *in patients with ASD.

Gene	Location/ nucleotide change	Amino acid change	Cases (n = 109)	Controls (n = 188)
*ASMT*	c.-376G>A	-	1	1

*ASMT*	c.-38C>T	-	1	0

*ASMT*	IVS5+2T>C	-	1	0

*MTNR1A*	c.-158C>T	-	1	0

*MTNR1B*	c.370G>A	V124I*	1	0

*MTNR1B*	c.-39GC>AA	-	1	0

* Predicted not to have any major effect on protein function, as assessed by Polyphen and SIFT.

The splice site mutation, IVS5+2T>C, and the 5'UTR variant in *ASMT *were identified in patients with autistic disorder and PDD-NOS, respectively. The promoter variant in *MTNR1A *was identified in a patient with PDD-NOS and the two *MTNR1B *mutations were identified in one patient with PDD-NOS (c.-39GC>AA) and one patient with autistic disorder (V124I). Interestingly, of the patients carrying variants that were not present in the unaffected group, only those with the splice site mutation in *ASMT*, and the non-synonymous V124I in *MTNR1B *met the diagnostic criteria for autistic disorder [additional file 3: Supplemental Table S3]. Moreover, four of the five patients carrying variants not present in controls have had seizures and/or diagnosed epilepsy and four of the five patients also displayed moderate to severe MR. For more detailed clinical information on the patients carrying the identified mutations, see additional file 3: Supplemental Table S3.

Common, previously reported, SNPs were identified at frequencies comparable to those found in the databases (data not shown). The two non-synonymous SNPs rs61747139 (K243R) in *MTNR1B*, and rs62620754 (S493N) in *GPR50 *were also genotyped in our reference group and the rare alleles of these polymorphisms were found to have similar frequencies in our non-patient cohort. Moreover, none of these variants were predicted to have any major effect on the encoded protein, according to computer based prediction.

## Discussion

In the present study, several rare variants were found in melatonin related genes in patients with ASD. Most importantly, the previously reported [15,19] splice site mutation, IVS5+2T>C, in *ASMT *was once again found in a patient with autistic disorder. To our knowledge, this mutation has now been found in six patients with ASD, two parents and only one control. Moreover, this variant has been shown to have a dramatic effect on melatonin synthesis, both in cultured cell lines and *in vivo *[15]. The other two variants identified in *ASMT *in our study were situated in upstream regulatory regions of the gene, and might therefore affect the expression of the *ASMT*-protein. This finding is in line with a recent study by Cai and coworkers [20], who found that a duplication in the *ASMT *gene was significantly more common in ASD, as compared to controls [20], thus suggesting that the expression of the ASMT protein may be altered in ASD patients. Moreover, three different transcripts of the ASMT gene have been identified

The identification of mutations in the genes encoding melatonin receptors suggests that also variants in other melatonin related genes might be implicated in the abnormal melatonin metabolism observed in ASD. Although the functional effects of the novel mutations were not assessed by laboratory assays in our study, bioinformatics analyses suggest that some of the variants identified indeed may influence the encoded protein. First, one of the novel variants identified, the V124I in the in the third transmembrane domain of the MTNR1B protein, is conserved through all vertebrates and among the three melatonin receptors, including GPR50 (figure 1). The most interesting of the identified variants were identified in patients for which we did not have access to DNA from parents and/or other relatives, hence, we do not know how these alleles are transmitted or whether they have appeared *de novo *in the patients. Further studies that screen ASD-patients for genetic variants in melatonin-related genes thus are highly warranted.

In summary, our identification of the functional splice site mutation in *ASMT *in yet another patient with autistic disorder support the notion that melatonin may be involved in the psychopathology of autism. Since melatonin regulates the rhythmic expression of clock genes in the hypothalamus [27], our results also indirectly support previous studies implicating clock genes in autism [22]. Although only one of the six mutations identified in our screen was present also in controls, our study sample is too small to conclude that mutations in melatonin related genes are enriched in patients with ASD. However, it cannot be excluded that some of the mutations found in melatonin related genes may infer an increased risk for circadian rhythm dysfunction in a subgroup of ASD patients. Moreover, the presence of seizures and moderate to severe MR in four of the five patients with the observed variants may suggest a broader role for melatonin related genes in neurodevelopmental disorders.

## Conclusions

Our findings suggest that melatonin related genes in general and *ASMT *in particular may be important candidates for further investigation in the search for genes involved in autism spectrum disorders and related neurobehavioral phenotypes. However, further studies of the novel variants identified in this study are warranted to shed light on their potential role in the pathophysiology of these disorders.

## Competing interests

The authors declare that they have no competing interests.

## Authors' contributions

LJ, EL, and CP carried out the molecular genetic studies, i.e., the sequencing and genotyping assays. LJ and EL together with JM and CP carried out the computer based sequence alignment for conservation studies, primer design, and identification of transcription factor binding sites. AB participated in the design of the molecular genetics experiments. MG, ML and KT recruited the patients. LJ, EL, and JM drafted the manuscript. JM designed the study, was involved in the data analysis, and edited the manuscript. All authors read and approved the final manuscript.

## Pre-publication history

The pre-publication history for this paper can be accessed here:

http://www.biomedcentral.com/1755-8794/3/10/prepub

## Supplementary Material

Additional file 1 **Supplementary Table S1. PCR primers and conditions used for sequencing in this study**. This table contains all primer sequences and PCR conditions used in the sequencing of the genes in this study. Click here for file

Additional file 2 **Supplementary Table S2. PCR primers used for genotyping in this study**. This table contains all primer sequences and PCR conditions used in the genotyping assays of this study. Click here for file

Additional file 3 **Supplementary Table S3. Clinical features of patients carrying mutations not observed in controls**. This table contains clinical information of the patients carrying mutations identified in this study. Click here for file
