# Supplementary material for: Mutation screening of melatonin-related genes in patients with autism spectrum disorders
Source: BMC Med Genomics. 2010 Apr 8;3:10. doi: 10.1186/1755-8794-3-10 (PMC3020629; doi:10.1186/1755-8794-3-10)
Supplement: Additional file 1 — Supplementary Table S1. PCR primers and conditions used for sequencing in this study. This table contains all primer sequences and PCR conditions used in the sequencing of the genes in this study. [file 1755-8794-3-10-S1.DOC]

**Supplementary Table S1.** PCR primers and conditions used for sequencing in this study

| **Gene/seq.** | **Amplicon**  **size (bp)** | **Name** | **Primer** | **Annealing temp. (°C)** |
| --- | --- | --- | --- | --- |
| ***MTNR1A*** |  |  |  |  |
| Promoter | 1014 | MTNR1ApromF2 MTNR1ApromR | TGGTCTGGCATAAAGTATATGAG ACAGGATGACCAGGAGGTTG | Td* 60-50 |
| Exon 1 | 578 | MTNR1Aex1F2 MTNR1Aex1R2(3) | ACGAGGAGAGGCCTCTGGGAC AGAACCAAGTGCTTGGGGAAG | Td* 60-50 |
| Exon 2 | 1239 | MTNR1Aex2F MTNR1Aex2R | AAACGGCCTCCACTTCATTT CTGAACCGCATTCCTCAGTTA | 60 |
| ***MTNR1B*** |  |  |  |  |
| Promoter | 804 | MTNR1BpromF MTNR1BpromR | TAACCCCTCCAAAAACCACA AGGAGCCGTTCTCTGACATC | 58 |
| Exon 1 | 516 | MTNR1Bex1F MTNR1Bex1R | CAAGTCCTCCTCCCATTCCTA GGAAAAGGCAGCGCATATC | 58 |
| Exon2 | 1450 | MTNR1Bex2F MTNR1Bex2R | TCGTGGAGGGTAGGATGTTC GTGTGTAGGGAAGGTCAATGG | 60 |
| ***GPR50*** |  |  |  |  |
| Promoter | 1121 | GPRpromF GPRpromR | CCGAGAGGAATCCTGACTGA AGAGAGCAAATGACAGCATCC | Td* 60-50 |
| Exon 1 | 729 | GPRex1F  GPRex1R | CAGTCGGAAGCAAGATGACA CACGGGAACGCTTTGTACTT | Td 60-50 |
| Exon 2 | 596 | GPRex2F1C GPRex2R1B | CCTGCACCTTAAATGTGTAGA CAGCAAGTTGGTTGTCAGGAT | 58 |
| Exon 2 | 705 | GPRex2F2 GPRex2R2 | GCTGTCCTGCCCAACATGTAC GGAGGATCTGGAATGGGGCTT | Td 60-50 |
| Exon 2 | 873 | GPRex2F3 GPRex2R3 | GGAAACCCCGATGAATGTCC CTGTGTCTAGATGCAGTAAGGC | Td* 60-50 |
| ***AA-NAT*** |  |  |  |  |
| Promoter/  Exon 1 | 996 | AA-NATpr+ex1F AA-NATpr+ex1R | CTGTGTGGGTAGGAGCAGAA CTCCACCTTCCAAGACACTTA | 65 |
| Exon 2/3/4 | 1758 | AA-NATex2+3+4F AA-NATex2+3+4R | GAATGTGCCCATTGATTTAGG AATGGGCTACTGTGAGGATG | 61 |
| ***ASMT*** |  |  |  |  |
| Promoter/  Exon 1B/C | 624 | ASMT1BF  ASMT1BR | AAAAGGGGTCTCACTATGTTGC  TGGAACGTGAGTGTGATGAAC | 58 |
| Exon 2 | 552 | ASMT2F  ASMT2R | TGGTGCAATCTCATTTGACTCTG  GGGTTCATGCCATTCTCCTG | 58 |
| Exon 3 | 950 | ASMT3F  ASMT3R | CAGCTGTACAAGGCAAGAGGA  CTTTCACCTCCTCCACTGCCA | 55 |
| Exon 4 | 283 | ASMT4F  ASMT4R | GCCTGGGCTACAGAGCTGAAA  CTCCTGGGTTGTGCCATTTG | 55 |
| Exon5 | 331 | ASMT5F  ASMT5R | CCTGTGGGGTATAGCTCCGTTC  CGCACATGTCAAAGCATCAGA | 64 |
| Exon 6 | 342 | ASMT6F  ASMT6R | AGCTTGCAGTGAGCGGAAATC  GCACCCATCGACTCGTCATTT | 64 |
| Exon 7 | 352 | ASMT7F  ASMT7R | TGGGTTGGACCCTTCATGAGT  GTGTTTCCGGGAGTGAGAGGA | 64 |
| Exon 8 | 338 | ASMT8F  ASMT8R | AGCCTGGAAGACCTGGGAAAG  CCTGTGGGATGATTTCAGTGC | 64 |
| Exon 9 | 506 | ASMT9F  ASMT9R | GGTGCCCTGACTGTCCTCTGA  CCATCAGCGTGGTCCTCAGTA | 64 |

All PCRs were performed with HotStarTaq polymerase (QIAGEN) on a GeneAmp PCR System 9700 (Applied Biosystems) at the following temperatures: 15 min at 95°C, 35 cycles of: 30 s at 95°C, 30 s at annealing temperature, 0.5-1 min at 72°C, followed by a final extension step of 10 min at 72°C. *PCRs performed with a Touch-down annealing temperature are indicated by td. Finally, to evaluate the PCR products, they were run onto an agarose gel (1,5%, stained with EtBr) and viewed under ultra-violet light to evaluate quantity and quality for further analysis.
