# Supplementary material for: Mutation screening of melatonin-related genes in patients with autism spectrum disorders
Source: BMC Med Genomics. 2010 Apr 8;3:10. doi: 10.1186/1755-8794-3-10 (PMC3020629; doi:10.1186/1755-8794-3-10)
Supplement: Additional file 2 — Supplementary Table S2. PCR primers used for genotyping in this study. This table contains all primer sequences and PCR conditions used in the genotyping assays of this study. [file 1755-8794-3-10-S2.DOC]

**Supplementary Table 2.** PCR primers used for genotyping in this study

| **Gene /SNP** | **Ampl.**  **Length**  **(bp)** | **Name** | **Primer** | **Label** |
| --- | --- | --- | --- | --- |
| *Pyrosequencing* |  |  |  |  |
| MTNR1A/  c.-158C>T | 179 | Mtnr1A_PSQ-158ct_2F  Mtnr1A_PSQ-158ct_2R  Mtnr1A_PSQ-158ct_2seq | GGTGCGGGGTTCGAGCTG  CCCGCCCGACCACTTGTT  CCGCGCCGGACGCCA ← | Biotin 5´ |
| MTNR1B/  c.370G>A | 53 | Mtnr1B_PSQ-370ga_fw  Mtnr1B_PSQ-370ga_rev  Mtnr1B_PSQ-370ga_seq | TTTGTGATGGGCCTGAGC  GCGATGGCAGTGATATTGAAG  GTGATGGGCCTGAGC → | Biotin 5´ |
| MTNR1B/  c.728A>G | 82 | Mtnr1B_PSQ-728ga_2fw  Mtnr1B_PSQ-728ga_2rev  Mtnr1B_PSQ-728gt_2seq | AAAGCCAAGCCAGAGAGCA  TTAGAAAGCTCCGCAAGTCG  CCGCAAGTCGCTGGG ← | Biotin 5´ |
| GPR50/ c.1478G>A | 88 | GPR50_PSQ_555gaF GPR50_PSQ_555gaR GPR50_PSQ_555gaSeq | CAACCCCAAGCCCATCAC GGGTTTAGGGTGGCTGGTG CACTCCAAGTCTGCCT | Biotin 5´ |
| ASMT/  c.-376G>A | 64 | ASMTpr_PSQ-376ct_fw (ga)  ASMTpr_PSQ-376ct_rev  ASMTpr_pyo-376ct_seq | ACCCAGTTTGCAGAAATTCG  TCTTATGGATTGAGCAGGTCCT  TTTGCAGAAATTCGC → | Biotin 5´ |
| ASMT/  c.-38C>T | 68 | ASMTpr_PSQ-38ct_fw  ASMTpr_PSQ-38ct_rev  ASMTpr_PSQ-38ct_seq | ACCTTGCCAGCAGGCTCTGT  AATCCAGCCGTGGCTTCC  TCCTTGAAGCAAGCG → | Biotin 5´ |
| *Fragment analysis* |  |  |  |  |
| GPR50 del 502-505 | 197 | GPR50fr_del502/505F GPR50fr_del502/505R | TTCATTTCAAGCCTGCTTCC  CTTAGGGTGGCTGGTAGTGG | 6-fam 5´ |

All PCRs were performed with HotStarTaq polymerase (QIAGEN) on a GeneAmp PCR System 9700 (Applied Biosystems) at the following temperatures: 15 min at 95°C, 35-40 cycles of: 30 s at 95°C, 30 s at annealing temperature, 0.5-1 min at 72°C, followed by a final extension step of 10 min at 72°C.
